# Transcriptome Analysis Provides Insights into Grain Filling in Foxtail Millet (*Setaria italica* L.)

**DOI:** 10.3390/ijms21145031

**Published:** 2020-07-16

**Authors:** Tao Wang, Hui Song, Pengtao Li, Yangyang Wei, Nan Hu, Zhenwen Chen, Weiqi Wang, Jinrong Liu, Baohong Zhang, Renhai Peng

**Affiliations:** 1 College of Biology and Food Engineering, Anyang Institute of Technology, Anyang 455000, China; wangtao6559@126.com (T.W.); lipengtao1056@126.com (P.L.); weiyangyang511@126.com (Y.W.); hnan2019@126.com (N.H.); ZhenwenChen@163.com (Z.C.); WeiqiWang@163.com (W.W.); 2 Innovation and Practice Base for Postdoctors, Anyang Institute of Technology, Anyang 455000, China; 3 Anyang Academy of Agriculture Sciences, Anyang 455000, China; songhui@163.com (H.S.); liujinrong63@163.com (J.L.); 4 Department of Biology, East Carolina University, Greenville, NC 27858, USA

**Keywords:** foxtail millet, grain filling, RNA-Seq, DEGs, starch biosynthesis

## Abstract

Grain filling is an importantly developmental process which is associated with the yield and quality of foxtail millet (*Setaria italic* L.). However, the molecular mechanisms of grain filling are rarely reported in foxtail millet. In our study, RNA-seq was performed to investigate the transcriptional dynamics and identify the key genes involved in grain filling in foxtail millet at five different developmental stages. A total of 11,399 differentially expressed genes (DEGs), including 902 transcription factors (TFs), were identified. Certain important genes involved in grain filling were discovered through a function annotation and temporal expression patterns analysis. These genes included genes associated with starch biosynthesis, cell-wall invertases, hormone signal transduction, and polyamine metabolism pathways. The expression levels of seven randomly selected DEGs were validated by a quantitative real-time polymerase chain reaction (qRT-PCR). This study provides the first insight into the changes in the gene expression of grain filling at different developmental stages in foxtail millet. These results could help understand the complex molecular mechanisms of the panicle formation in foxtail millet and other cereal crops.

## 1. Introduction

Kernel weight plays a vital role in the yield of cereal crops and is determined by the duration and rate of grain filling. Therefore, improving grain filling increases the grain weight and cereal yield [1,2]. Starch is the major ingredient of the kernels, and starch accumulation is a complex process that requires many enzymes. A low activity of crucial enzymes participating in sucrose-to-starch conversion within the kernels has been proven to inhibit the grain filling process [3,4]. The change in plant hormone levels could also affect the grain filling process in plants. Increasing ethylene levels in developing seeds inhibits the accumulation and activities of starch synthesis-related enzymes, and further inhibits the biosynthesis of carbohydrates in the developing spikelets and decreases the grain-filling rate [5,6]. Abscisic acid (ABA) also regulates the sink activity during grain filling [7]. Additionally, polyamines significantly promote the grain filling of inferior grain in wheat [8]. Poor filling in the inferior spikelets is mainly due to the lower auxin (IAA) concentrations in rice grains [9]. 

Several pivotal genes involved in grain filling have been identified; their molecular pathways have also been deciphered in crops. The GRAIN INCOMPLETE FILLING 1 (*GIF1*) gene is required for carbon allocation during early grain-filling in rice and encodes a cell-wall invertase. The overexpression of *GIF1* increased the grain production in rice [10]. SWEETs also affect the sugar allocation in plants. Maize mutants *ZmSWEET4c* and *OsSWEET4* are defective in seed filling [11]. The GRAIN-FILLING RATE1 (*GFR1*) gene enhanced the expression of Rubisco genes in the Calvin cycle, which in turn promoted the synthesis of sucrose and increased the grain-filling rate and yield in rice [12]. *GF14f* is a member of the 14-3-3 protein family which could negatively affect the grain filling of inferior spikelets in rice through inhibiting the enzyme activity involved in sucrose breakdown, starch synthesis, the tricarboxylic acid (TCA) cycle, and glycolysis. [13]. Additionally, microRNAs (miRNAs) play an important role during plant growth and development, including grain filling [14,15]. A recent study showed that *miR1432* regulated the grain filling in rice through targeting the expression of *OsACOT* [16]. With the development of high-throughput sequencing technology, transcriptome sequencing has become an effective approach to study the dynamic changes in gene expression during grain filling in different plant species [17,18,19,20].

Foxtail millet (*Setaria italica* L.), which originated in China, is one of the oldest domesticated diploid C4 Panicoid crops. Foxtail millet is an important food and feed crop in arid and semi-arid regions of Asia and Africa, especially in China and India [21]. Recently, foxtail millet has become an ideal model plant for the study of C4 monocots due to the factors that foxtail millet has a small genome size, lower repetitive DNA, an inbreeding nature, and a short life cycle [22]. The whole genome sequencing of foxtail millet promoted more research in the field of molecular biology and functional genomics in this crop [23,24,25]. Although some progress has been made on the grain development in foxtail millet [26], the molecular genetics-related research is still very weak compared with other cereal crops; the molecular mechanism and regulatory network of grain filling have not been reported in foxtail millet.

In this study, to gain insight into the transcriptional dynamics and identify the crucial genes involved in grain filling, we employed high-throughput deep sequencing technology to perform a comprehensive transcriptomic analysis of foxtail millet at five different developmental stages, including at 7, 14, 21, 28, and 35 days after anthesis (DAA). The results in this study will provide new insights into the molecular mechanism of foxtail millet grain filling and yield.

## 2. Results

### 2.1. Dry Weight Change During Foxtail Millet Grain Filling

**Figure 1:**
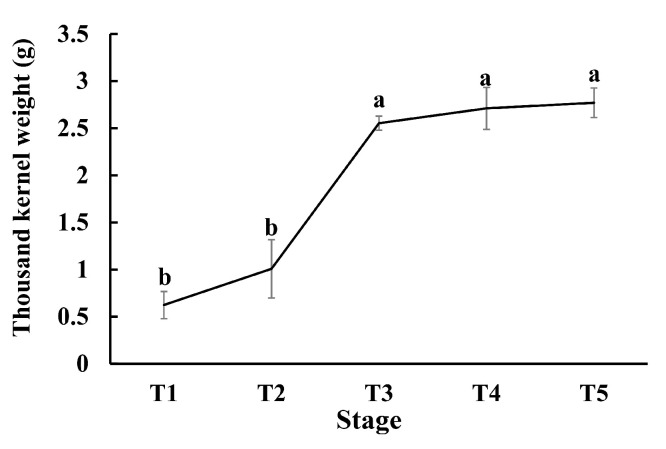
The change in thousand kernel weight (TKW) in different stages during grain filling. Error bars represent ± the standard error of the mean (*n* = 3, n represents the biological replicates). Different letters indicate significant differences at the 0.05 level.

To investigate the dynamic changes in kernel weight during grain filling, spikelets were harvested on the 7, 14, 21, 28, and 35 DAA, corresponding to T1, T2, T3, T4, and T5, throughout the grain filling. The TKW (thousand kernel weight) changes were significant as the time went on (*p* < 0.05). The TKW was gradually increased from T1 to T5, and the most significant change was between T2 and T3. After T3, the kernel weight change was not significant (Figure 1).

### 2.2. Overview of Transcriptome Data During Grain Filling in Foxtail Millet

To gain insight into the dynamic changes in gene expression during grain filling in foxtail millet, 15 libraries were constructed for RNA-seq from five developmental stages (three biological replicates for each developmental stage). The detailed sequencing data are shown in Table 1. A total of 786.11 million raw reads were obtained. After removing the adaptor sequences and low-quality sequence reads, 764.55 million clean reads were obtained, with an average of 50.97 million clean reads per library. Approximately 90.13% to 96.31% of the reads were mapped to the foxtail millet reference genome, and 85.36% to 93.47% of the reads could be mapped uniquely to the foxtail millet reference genome. Q30 of the clean reads of the 15 libraries were above 91.41%, indicating the high reliability of the data.

**Figure 2:**
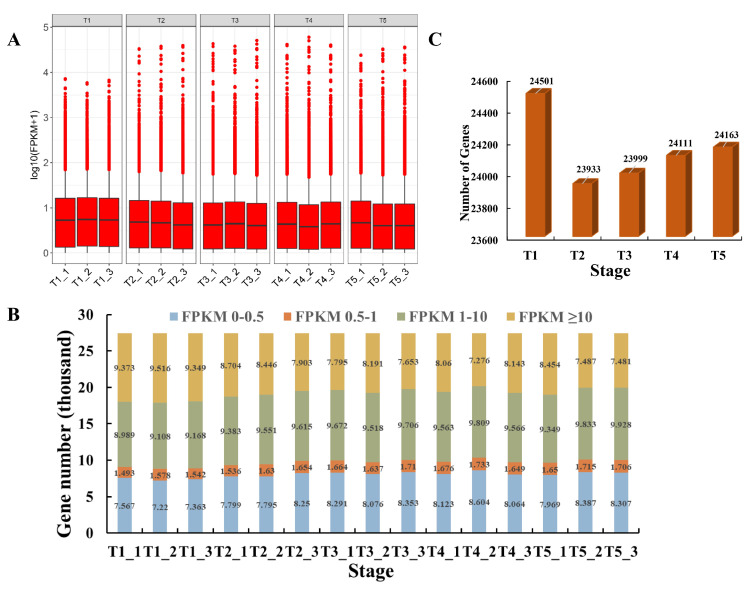
Gene expression levels during different developmental stages in foxtail millet, generated using high-throughput deep sequencing technology. (**A**) Expression density of genes in different developmental stages. (**B**) Distribution of genes with different expression levels at each developmental stage. (**C**) Total number of expressed genes at each developmental stage.

All the 27,422 identified genes were expressed with different expression patterns during the different developmental stages in foxtail millet (Figure 2a, Table S1). These genes were divided into four groups based on their expression level (Figure 2b). The number of genes with Fragments Per kb Per Million Reads (FPKM) < 10 was increased gradually from T1 to T5. In contrast, the highly expressed (FPKM ≥ 10) genes decreased gradually during grain filling. Not all of the 27,422 identified genes were always expressed in all the developmental stages. Compared with the T1 developmental stage, there were less genes expressed in the other stages, especially the T2 and T3 developmental stages (Figure 2c).

### 2.3. Identification and Analysis of Differentially Expressed Genes (DEGs)

**Figure 3:**
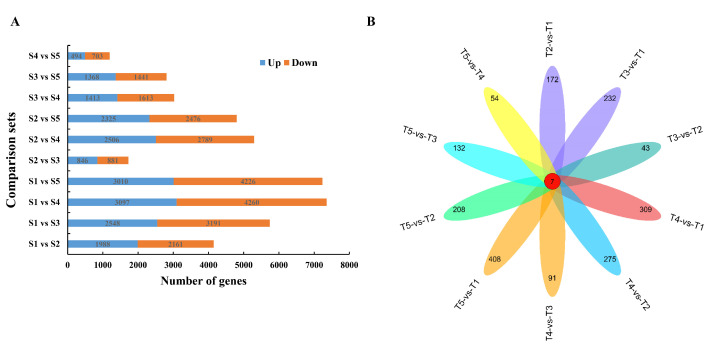
Numbers of specific differentially expressed genes (DEGs) in different comparison groups during the grain filling. (**A**) Numbers of up- and down-regulated DEGs in different comparison groups. (**B**) Venn diagram for unique DEGs in different comparison groups.

A total of 11,399 DEGs were identified; the expression level of each gene was compared among the five developmental stages (Figure 3). A Venn diagram was created to identify the unique differentially expressed genes (DEGs) of each comparison. As shown in Figure 3b, a comparison of the DEGs among the 10 sets showed that 172, 43, 91, and 54 unique DEGs were identified in the T1 vs. T2, T2 vs. T3, T3 vs. T4, and T4 vs. T5 comparison sets, respectively, and 7 DEGs were contained in all comparison sets. 

In order to further analyze the DEGs, a Gene Ontology (GO) classification analysis of the identified unique DEGs was performed (Figure S1). All the DEGs were divided into the biological process, cellular component, and molecular function categories. Cellular process, metabolic process, single-organism process, response to stimulus, and biological regulation were the top five classes in the biological process. Cell, cell part, organelle, membrane, and membrane part were the top five classes in the cellular components. Binding, catalytic activity, nucleic acid binding transcription factor activity, transporter activity, and enzyme regulator activity were the top five classes in the molecular functions. 

When subjected to a Kyoto Encyclopedia of Genes and Genomes (KEGG) pathway analysis, the significantly enriched 20 pathways are shown in Figure S2. 

### 2.4. Analysis of the Temporal Expression Patterns of DEGs

**Figure 4:**
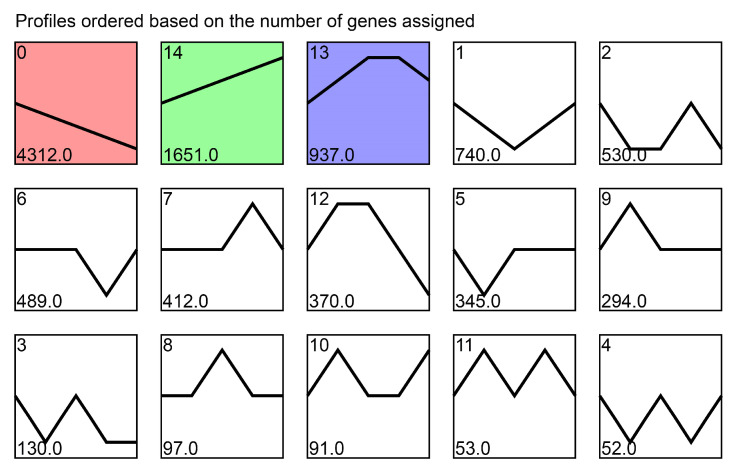
The expression trend analysis of DEGs during grain filling from T1 to T5 in foxtail millet. The top number represents the profile category. The bottom number represents the total number of genes in each category. The line represents the gene expression trends of each profile.

**Figure 5:**
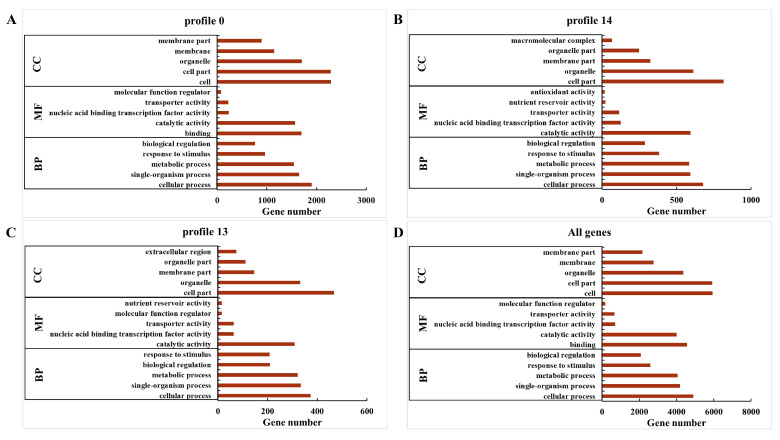
Top five Gene Ontology (GO) classification in each category of DEGs in (**A**) profile 0, (**B**) profile 14, (**C**) profile 13, and (**D**) all genes during the grain filling from T1 to T5. The Y-axis represents the GO term of genes, and the X-axis represents the number of genes. BP represents biological process, MF represents molecular function, and CC represents cellular component.

In order to explore the temporal expression patterns of the identified DEGs during grain filling in foxtail millet, gene expression profile clustering was employed. The expression trends of all 11,399 DEGs were sorted into 15 profiles (Figure 4). Each profile represents a set of genes with a similar expression pattern during grain filling. The top three expression profiles of the DEGs were profile 0 (4312 genes, 37.83%), profile 14 (1651 genes, 14.48%), and profile 13 (937 genes, 8.22%). To identify the putative functions of the DEGs in these three profiles, a GO classification analysis was performed with all the genes and DEGs in these three profiles (Supplementary Data Set 1). All the genes were divided into three categories, including biological process, cellular component, and molecular function. The top five GO terms in each category are shown in Figure 5; the compositions of the biological processes were the same, but the compositions of the cellular components and molecular functions were different between profile 0, profile 14, profile 13, and all genes.

**Figure 6:**
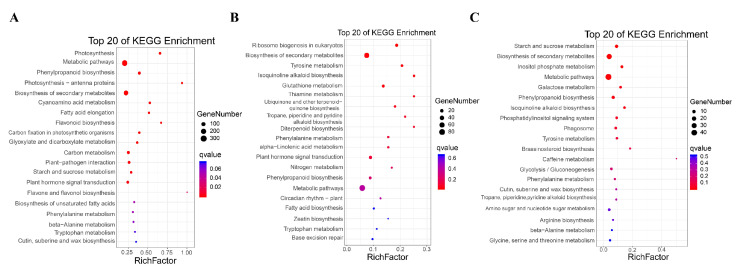
The top 20 enriched Kyoto Encyclopedia of Genes and Genomes (KEGG) pathways of DEGs in (**A**) profile 0, (**B**) profile 14, and (**C**) profile 13 during the grain filling from T1 to T5. The Y-axis represents the pathway of genes, and the X-axis represents the Rich Factor.

When subjected to a KEGG pathway analysis, the significantly enriched 20 pathways of profile 0, profile 14, and profile 13 are shown in Figure 6. In profile 0, the photosynthesis, metabolic pathways, phenylpropanoid biosynthesis, photosynthesis-antenna proteins, and biosynthesis of secondary metabolites were the five dominant pathways (Figure 6a). In profile 14, the ribosome biogenesis in eukaryotes, biosynthesis of secondary metabolites, tyrosine metabolism, isoquinoline alkaloid biosynthesis, and glutathione metabolism were the five dominant pathways (Figure 6b). In profile 13, the starch and sucrose metabolism, biosynthesis of secondary metabolites, inositol phosphate metabolism, metabolic pathways, and galactose metabolism were the five dominant pathways (Figure 6c).

### 2.5. DEGs Are Involved in Starch Biosynthesis

**Figure 7:**
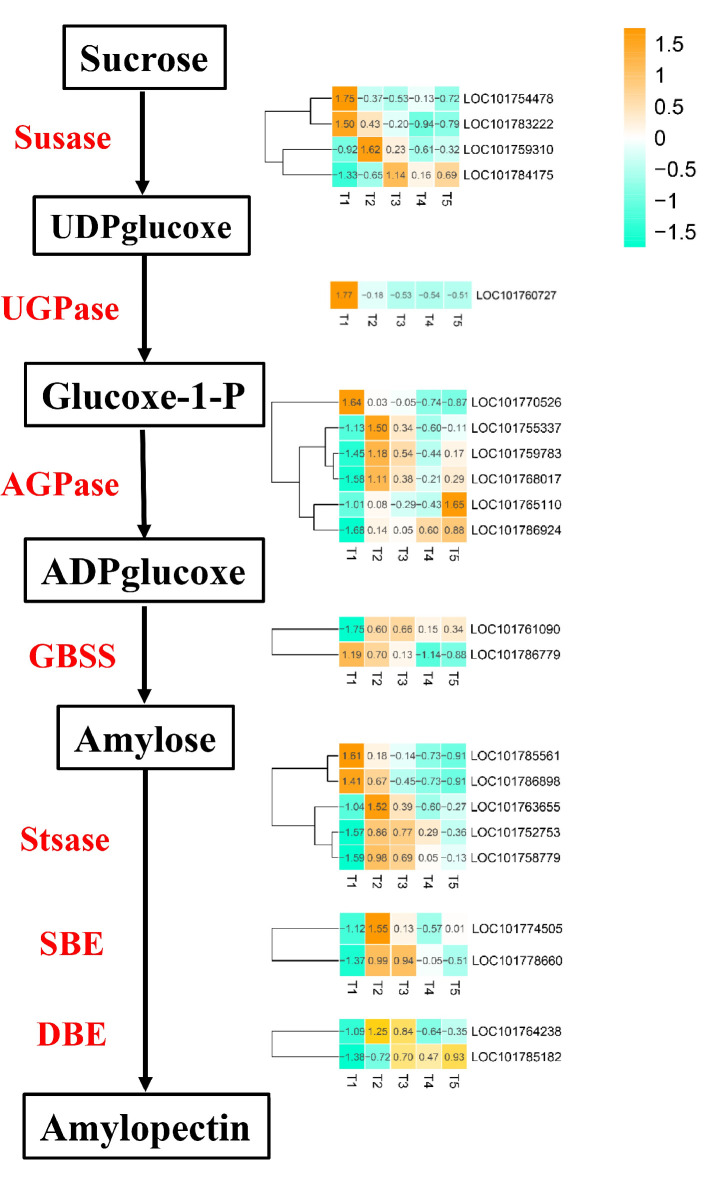
Heatmap analysis of genes associated with sucrose-starch conversion during grain filling from T1 to T5. SUS: Sucrose synthase; UGPase: UDP glucose pyrophosphorylase; AGPase: ADP glucose pyrophosphorylase; GBSS: Granule-bound starch synthase; SS: Starch synthase; SBE: Starch-branching enzyme (SBE); DBE: Debranching enzyme. Changes in expression level are indicated by a change in color; light green indicates a lower expression level, whereas orange indicates a higher expression level.

The conversion of sucrose to starch is the key process that affects grain filling in crops. In this study, we identified 22 DEGs associated with starch and sucrose metabolism (Figure 7), which include four sucrose synthase (SuSase), one UDP-glucose pyrophosphorylase (UGPase), six adenosine diphosphate glucose pyrophosphorylase (AGPase), two starch glucosyltransferase/granule-bound starch synthase (GBSS), five starch synthase (StSase), two starch-branching enzyme (SBE), and two isoamylase/debranching enzyme (DBE). Of these, two SuSase, one UGPase, one AGPase, one GBSS, and two StSase genes were expressed with the highest levels at the T1 stage and then declined gradually. One SuSase, three AGPase, three StSase, two SBE, and one DBE genes were expressed with the highest levels at the T2 stage. One SuSase gene and one GBSS gene were expressed with the highest levels at the T3 stage. Two AGPase and one DBE genes were expressed with the highest levels at the T5 stage.

### 2.6. DEGs Are Involved in Cell-Wall Invertases

**Figure 8:**
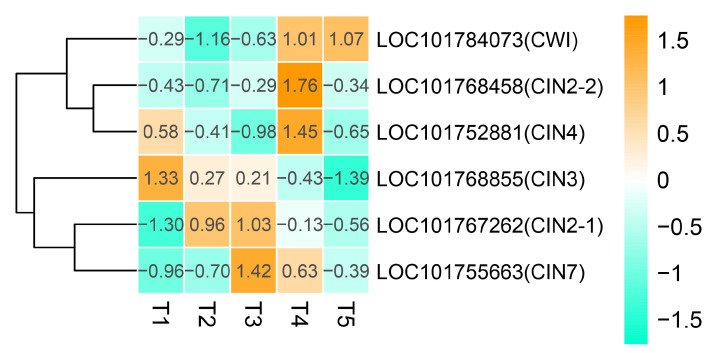
Heatmap analysis of genes associated with cell-wall invertases during grain filling from the T1 to T5 stage. Changes in the expression levels are indicated by a change in color; light green indicates a lower expression level, whereas orange indicates a higher expression level.

Cell-wall invertases (CIN) play important functions in the grain filling process. A total of 12 cell-wall invertases (CIN) genes were identified, and six of them were differentially expressed during different developmental stages (Figure 8). The expression levels of LOC101767262 (*CIN2-1*) and LOC101755663 (*CIN7*) increased gradually and reached the highest at T3, then declined. LOC101752881 (*CIN4)* exhibited a relatively high expression level at T1 and started to decline at T2 and T3 but increased suddenly and reached a peak expression at T4. LOC101768855 (*CIN3*) exhibited the highest expression level at T1, then declined gradually. The expression levels of LOC101768458 (*CIN2-2*) and LOC101784073(*CWI*) gradually increased during grain filling and reached the peak at the T4 and T5 stages, respectively. LOC101767262 (*CIN2-1*) and LOC101768458 (*CIN2-2*) have similar coding sequences. Their different expression may be due to other regulatory elements relating to these two genes, such as promoter.

### 2.7. DEGs Are Involved in Polyamine Metabolism Pathways

**Figure 9:**
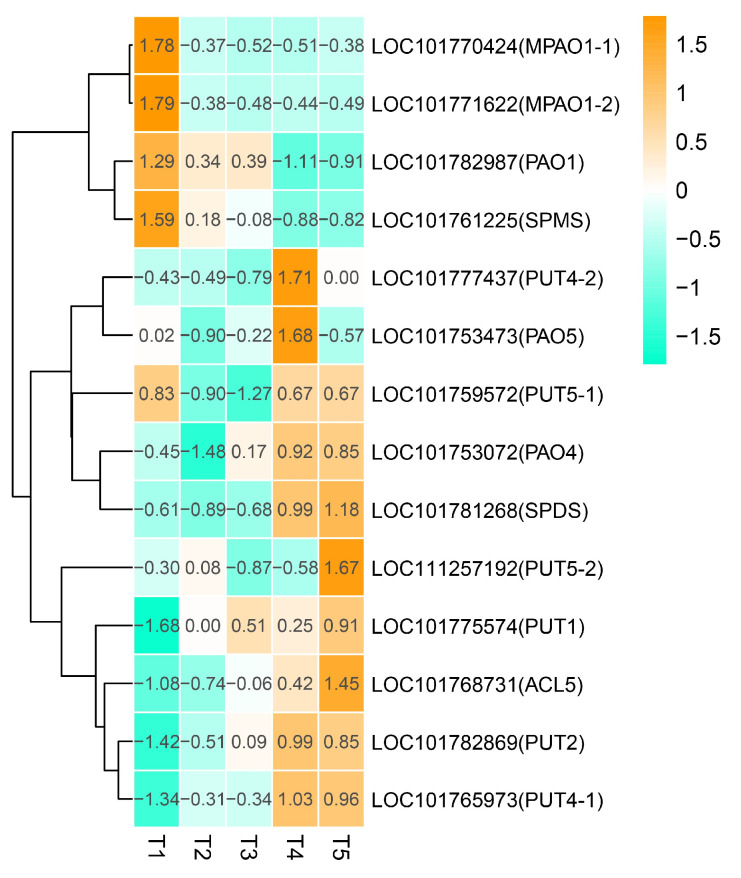
Heatmap analysis of genes associated with polyamine metabolism during grain filling from the T1 to T5 stages. Changes in expression levels are indicated by a change in color; light green indicates a lower expression level, whereas orange indicates a higher expression level.

Polyamines, as intracellular messengers, are closely related to the grain development and grain filling. A total of 14 DEGs were identified and are associated with polyamine metabolism pathways. These 14 polyamines included five polyamine oxidase (PAO), six polyamine transporter (PUT), one thermospermine synthase (ACL5), one spermine synthase (SPMS), and one spermidine synthase (SPDS) (Figure 9). Three PAOs and one SPMS showed the highest expression at the early stage of grain filling (T1), then declined gradually. The expression of PAO4, PAO5, PUT2, and two PUT4s exhibited relatively low expression levels at the early stages of grain filling, then increased gradually, and finally reached a peak at the T4 stage. A similar expression pattern was also found in the PUT1, PUT5-2, ACL5, and SPDS genes, but their expression peak was observed at the T5 stage. In addition, PUT5-1 exhibited a relatively high level at T1, T4, and T5, but a relatively low level at the T2 and T3 stages. Sequence differences in the non-coding regions may lead to the different expression patterns of LOC101765973 (PUT4-1)/LOC101777437 (PUT4-2) and LOC101759572 (PUT5-1)/LOC111257192 (PUT5-2).

### 2.8. DEGs Are Involved in Hormone Signal Transduction Pathways

Hormone signals play an important role in plant growth and development. In our results, 108 DEGs were associated with plant hormone signal transduction, including the ABA signaling pathway (14 genes), auxin signaling pathway (54 genes), brassinolide (BR) signal transduction pathway (1 genes), cytokinin (CTK) pathway (14 genes), ethylene (ETH) pathway (14 genes), jasmonic acid (JA) pathway (9 genes), and salicylic acid (SA) pathways (2 genes) (Table S2).

Gene expression profile clustering was also employed to explore the temporal expression patterns of these genes associated with plant hormone signal transduction during the grain filling in foxtail millet (Figure S3). The majority of plant hormone-related genes belonged to the category of profile 0 (38/108, 35.18%), which exhibited a gradually reduced expression pattern. The expression of 15.74% (17/108) of genes was up-regulated during grain filling in foxtail millet (profile 14).

### 2.9. Many TFs Are Differentailly Expreesed during Grain Filling in Foxtail Millet

**Figure 10:**
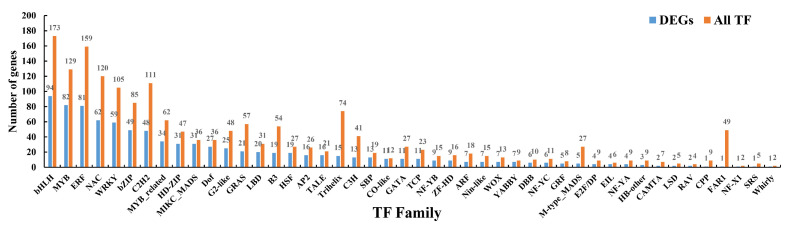
The distribution of transcription factor families with differential expression during grain filling in foxtail millet. The Y-axis represents the number of genes, and the X-axis represents the transcription factor (TF) families.

The expression dynamics of TF genes were investigated during grain filling. A total of 902 TFs, belonging to 46 families, were expressed in at least one tested stage (Supplementary Data Set 2). The top five largest TF families were basic helix-loop-helix (bHLH) (94), v-myb avian myeloblastosis viral oncogene homolog (MYB) (82), ethylene responsive factor (ERF) (81), NAM-ATAF1-2-CUC2 (NAC) (62), and WRKY domain transcription factors (WRKY) (59) (Figure 10). Gene expression profile clustering was employed to explore the temporal expression patterns of these TFs during grain filling in foxtail millet (Figure S4). The top four profiles of TF were same as those of all the DEGs; the latter profiles had some differences between TF and all the DEGs. Most TFs belonged to the category of profile 0 (197/902, 35.59%), which was gradually down-regulated during grain filling in foxtail millet. However, the expression of 16.19% (146/902) TFs was up-regulated during grain filling in foxtail millet (profile 14).

### 2.10. Verification of RNA-Seq Gene Expression by qRT-PCR

A qRT-PCR was performed to verify the reliability of the RNA-seq data. Seven DEGs were randomly selected, including LOC101752982, LOC101753161, LOC101753406, LOC101754192, LOC101754725, LOC101755337, and LOC101758382. The expressions of these DEGs were consistent with the results of the RNA-seq data (Figure S5), confirming the reproducibility of the RNA-seq data.

## 3. Discussion

Grain filling is the second phase of kernel development, which is marked by a rapid weight gain in kernels as a result of quick starch accumulation. The grain filling not only influences kernel size, but also the crop yield and quality [2]. Improvement of grain filling, especially the grain filling rate, has been proved as an effective way to increase the yield and quality of crops [27]. The formation of kernels has been studied for a long time, but research on the molecular mechanisms of kernel formation has only been performed in recent years, especially in model plants [28]. Many quantitative trait loci (QTLs) associated with the kernel size and grain filling rate have been identified in crops [29,30]. Numerous genes associated with grain filling have also been identified and cloned in rice and maize [11,12,31,32,33].

In our study, we found that the kernel weight was rapidly increased between the T2 and T3 stages. A comparison of the DEGs showed that the highest number of DEGs existed between the T2 and T1 stages, suggesting that the gene expression levels changed dramatically at the T2 stage in foxtail millet spike. Therefore, we believe that the T2 stage is the pivotal stage during grain filling in foxtail millet.

It is important for grain filling to convert sucrose to starch. The enzymes involved in this process, such as for sucrose degradation and starch synthesis, are considered to be key [20,34]. These enzymes include SuSase (EC 2.4.1.13), AGPase (EC 2.7.7.27), StSase (EC 2.4.1.21), SBE (EC 2.4.1.18), UGPase (EC 2.7.7.9), DBE (EC 3.2.1.68), and GBSS (EC 2.4.1.242) [18,35]. In this study, we identified 22 differentially expressed genes that encoded enzymes associated with starch and sucrose metabolism. These genes included four SuSase, one UGPase, six AGPase, two GBSS, five StSase, two SBE, and two DBE. SuSase promoted the breakdown of sucrose in grains, which led to an increased gradient between the source and the sink, and further enhanced the transport of sucrose from the phloem to the grains [36]. The three SuSase showed the highest expression at T1, T2, and T3. In addition, the expression of the other six key enzymes involved in starch synthesis showed different expression patterns. These results indicate that these genes play key roles in grain filling through the enhancement of their enzyme activities and starch accumulation at different grain filling stages.

Several studies have indicated that the members of the cell wall invertase (CIN) gene family play an important role in grain filling. In rice, *OsCINs* regulated sucrose partitioning to the embryo and endosperm and played important roles in grain filling [37,38]. *GIF1*, encoding a cell wall invertase (*OsCIN2*), is required for carbon partitioning through the regulation of sucrose transport and unloading during the early grain-filling stage. The overexpression of *GIF1* increased the grain production in rice [10]. In our study, six *CIN* genes were identified as DEGs during foxtail millet grain filling. Among them, CIN3 exhibited the highest expression level at T1, then declined gradually. This suggests that CIN3 may play an important role at the early stage of grain filling. The expression levels of CWI, *CIN2-2,* and *CIN4* increased gradually and reached the highest at T4 and T5, respectively, indicating that they mainly regulate the grain filling process at a later stage.

In crops, kernel development and grain filling are regulated by plant hormones. The ABA level was positively correlated with the grain filling rate [39]. Auxin could induce the expression of growth-regulating genes to regulate the panicle and spikelet development in rice [40]. Some studies showed that cytokinin is involved in regulating grain production [41,42]. Other plant hormones, including BR, ET, JA, and SA, also play a crucial role in seed development and grain production [5,17,18,43,44,45]. In this study, we observed that many differentially expressed genes were related to plant hormone biosynthesis and/or signaling pathways; these genes included *PYLs* (abscisic acid receptor), *ABI5* (ABSCISIC ACID-INSENSITIVE 5), *PP2C* (probable protein phosphatase 2C), *SAPKs* (serine/threonine-protein kinase), *SAURs* (auxin-responsive protein), *JAR* (jasmonic acid-amido synthetase), *EIN2* (ETHYLENE-INSENSITIVE 2), and *TIFY* (Table S2). Many of them were expressed at a high level at the early stage, indicating that these genes might be involved in early grain filling events through activating the expression of downstream-related genes. LOC101766228 (*PP2C*), LOC101772577 (*ABI5*), LOC101759755 (*ARF2-like*), and other genes increased gradually and reached the highest at the T3, T4, and T5 stages, indicating that they mainly regulate the grain filling process at a later stage (Table S2).

Except for plant hormones, endogenous plant growth regulators such as Polyamines (PAs) also regulate grain filling in cereals. High levels of spermidine and spermine promoted grain filling in rice [46,47,48,49]. Putrescine is the central product of the polyamine biosynthesis pathway. Permidine synthetase (SPDS) catalyzed putrescine to form spermidine, and then spermine synthases (SPMS) catalyzed spermidine to spermine [50]. In monocotyledon, polyamine oxidase (PAO) is mainly responsible for the catabolism of polyamines [51]. Different PAOs have different preferences for substrates, *OsPAO3* favored spermidine as a substrate, followed by thermospermine and spermine; however, *OsPAO4* and *OsPAO5* preferred spermine and thermospermine, but not spermidine [52]. In our study, *SPMS* showed the highest expression at the early stage of grain filling (T1), then declined gradually, indicating that spermine may play a main function at the early stage of grain filling. In contrast, thermospermine synthase *ACL5* and *SPDS* expressed at a relatively low level at the early stages of grain filling, then increased gradually and reached the peak at T5 stage, indicating that spermidine and thermospermine may play an important role at the later stage of grain filling in foxtail millet. Three *PAO1* genes have a similar expression pattern with *SPMS*, indicating that *PAO1* preferred spermidine but not spermine in foxtail millet. However, *PAO4* and *PAO5* may prefer spermine but not spermidine and thermospermine, because it has a similar expression pattern to *SPDS* and *ACL5.* In addition, six polyamine transporters showed a higher expression level at the later developmental stages (T4 and T5), suggesting that they may play an important role at a later stage during grain filling in foxtail millet.

TFs are the pivotal regulatory proteins in grain filling, such as NF-YB1 (Nuclear Factor Y B1) and bZIP (basic leucine zipper) [53,54]. In our study, multiple TF families, including bHLH, MYB, ERF, NAC, and WRKY, were differentially expressed during grain filling in foxtail millet. In addition, some TF families on the list are in accordance with the general TF family abundance in the foxtail millet genome, such as bHLH (94/173), MYB (82/129), and ERF (81/159). Some TF families have more members in the foxtail millet genome, but less belong to DEGs in this study, such as C2H2 (48/111) and FAR1 (1/49). Some TF families ranked high in seed but were not abundant in the foxtail millet genome, such as MIKC-MADS (31/36) and HD-ZIP (31/47), suggesting that these TF families may play an important role during grain filling. Many TFs exhibited a pattern with gradually decreased expression levels, suggesting that they may play a negatively regulatory role during grain filling in foxtail millet. Meanwhile, the expression of 16.19% of TFs keeps rising during grain filling, suggesting that these TFs may be positive correlated with grain filling in foxtail millet.

In conclusion, in this study we provided insight into the transcriptional dynamics during grain filling at five different developmental stages in foxtail millet. The DEGs and their differential expression pattern were validated by qRT-PCR during grain filling in foxtail millet. Some DEGs were expressed at the early stage (T1 and T2), and some genes had a higher expression level at the later stage, indicating that different DEGs may play different roles during grain filling in foxtail millet. These results provide useful information for exploring the molecular mechanism of grain filling in foxtail millet and also provide a solid theoretical foundation for foxtail millet improvement.

## 4. Materials and Methods 

### 4.1. Plant Materials and Sample Collection

The seeds of foxtail millet (*Setaria italic* L.) cv. “Yu Gu 18” were obtained from Anyang Academy of Agriculture Sciences (Anyang, Henan, China). Foxtail millet was planted and grown in the experimental station of Anyang Institute of Technology (Anyang, Henan, China). A randomized block design was used to perform the field experiments, with 3 m × 3 m for each plot. The soil was yellow loam and contained the following nutrients: organic matter (1.31%), available nitrogen (54.6 μg/g), available phosphorus (20.4 μg/g), and available potassium (212 μg/g). Regular agronomical practices were performed in the field. The same-aged spikelets with the same size were harvested at the 7, 14, 21, 28, and 35 DAA, which corresponded to T1, T2, T3, T4, and T5, respectively. Five spikelets from the same field plot were mixed together as one biological replicate. Three biological replicates were selected for each developmental stage from three different field plots. All the samples were immediately frozen in liquid nitrogen and stored at −80 °C for later RNA extraction and RNA-Seq. 

For biomass analysis, 10 spikelets were harvested at the 7, 14, 21, 28, 35 DAA, which corresponded to T1, T2, T3, T4, and T5, respectively. The collected spikelets were first dried in an oven (105 °C) for 30 min, followed by moving to another drying oven (70 °C) until the weight of the spikelets was constant. The dried spikelets were threshed separately, and then 100 seeds were selected for each spikelet and the TKW was weighted and calculated.

### 4.2. RNA Extraction, Library Construction, and Sequencing

The total RNAs were extracted according to our previous studies [55,56] using the mirVana miRNA Isolation Kit (Ambion, Inc., Austin, TX, USA). RNase-free DNase (Promega, Madison, Wisconsin, USA) was added into the RNA samples to remove the genomic DNA contamination. The quality of the RNAs was evaluated using the Agilent 2100 Bioanalyzer (Agilent Technologies, Santa Clara, CA, USA). The total RNAs (4 μg per sample) were used to purify and fragment the mRNAs with RNA purification beads. DNA library construction and RNA-Seq high-throughput sequencing were commercially performed by Shanghai OE Biotech (Shanghai, China). A total of 15 qualified libraries were sequenced on the Illumina HiSeq 2500 sequencing platform (Illumina, San Diego, CA, USA) with 125 paired-end sequencing. The raw sequencing data were deposited into the public databases of the National Center for Biotechnology Information (NCBI) (BioProject accessions: PRJNA633007; BioSample accessions: SAMN14930334).

### 4.3. Quality Control and Sequence Mapping 

The publicly available software Trimmomatic version0.36 [57] was used to process the raw data generated from the deep sequencing. Clean reads were obtained by removing the sequences with low quality. Then, hisat2 version2.2.1.0 [58] was employed to map the clean reads to the foxtail millet reference genome [23].

### 4.4. Identification of Differentially Expressed Genes (DEGs)

The publicly available software cufflinks version2.2.1 [59] was employed to calculate the expression level of each identified gene. The value of gene expression was presented by the Fragments Per kb Per Million Reads (FPKM). Differentially expressed genes were identified using the software DESeq version1.18.0 [60] with the R package functions estimateSizeFactors and nbinomTest. For a specific gene, if the fold change was > 2 or the fold change was < 0.5 with a *p* value < 0.05, this gene was identified as a DEG.

### 4.5. Gene Function Analysis and Enrichment

A Gene Ontology (GO, http://www.geneontology.org/, accessed on 1st of January 2020) [61] and functional enrichment analysis were performed on the same set of DEGs. Finally, a Kyoto Encyclopedia of Genes and Genomes (KEGG) analysis was employed to map all the DEGs to a specific pathway in the database (http://www.genome.jp/kegg/pathway.html, accessed on the 21st of November 2019) [62] by the Blast_v2.2.26 software. A hyper geometric distribution test was also carried out to identify the GO functions and KEGG pathways in which the DEGs were significantly enriched (*p* value < 0.01).

### 4.6. Temporal Expression Patterns of DEGs

The Online Omicshare Tools (https://www.omicshare.com/tools/Home/Soft/trend, accessed on the 2nd of November 2020) were employed to analyze the temporal expression patterns of the selected DEGs. The gene expression patterns were based on Log_2_FPKM. The program was performed with the default settings, except for the following setting: the *p* value of < 0.05 and fold change >2.

### 4.7. Quantitative Real-Time PCR Analysis

To validate the reliability of gene expression generated by the RNA-seq, 7 DEGs were randomly selected and tested. Briefly, the total RNAs were isolated from the foxtail millet leaves using the TRIzol reagent (Tiangen, Beijing, China) according to the manufacturer’s instructions. A FastKing RT kit (Tiangen, Beijing, China) was employed to synthesize the first-strand cDNA. A quantitative real time PCR (qRT-PCR) was performed using the SYBR Green PCR Master Mix Reagent (SuperReal PreMix Plus, Tiangen, China) on an ABI 7500 (Applied Biosystems, Foster City, CA, USA). The changes in gene expression were determined by the 2^−ΔΔCt^ method [63]. The foxtail millet Actin 7 (AF288226.1) gene was used as a reference gene. Three biological and three technical replicates were run for each gene. The primers for qRT-PCR used in the gene expression analysis were listed in Table S3.

### 4.8. Statistical Analyses

A one-way ANOVA was used to analyze the data followed by least significant difference multiple comparison tests (*p* < 0.05) using SPSS ver. 18.0 (SPSS, Chicago, IL, USA).

## Figures and Tables

**Table 1:** Overview of the RNA-seq data.

Sample	Raw Reads (M)	Clean Reads (M)	GC Content (%)	Q30 (%)	Mapped Reads (M (%))	Unique Mapped Reads (M (%))	Multiple Mapped Reads (M (%))
T1_1	57.63	55.89	53.16	91.57	53.66 (96.00)	52.24 (93.47)	1.41 (2.53)
T1_2	53.17	51.58	53.35	91.54	49.49 (95.95)	48.19 (93.43)	1.30 (2.52)
T1_3	54.00	52.45	53.50	91.41	49.92 (95.19)	48.64 (92.74)	1.29 (2.45)
T2_1	49.95	48.59	53.18	91.71	46.71 (96.13)	44.81 (92.21)	1.91 (3.92)
T2_2	50.56	48.95	53.41	92.08	46.78 (95.56)	44.73 (91.38)	2.05 (4.18)
T2_3	54.59	52.87	53.37	91.88	50.92 (96.31)	48.60 (91.93)	2.32 (4.38)
T3_1	49.44	47.89	53.87	92.03	45.35 (94.70)	43.35 (90.52)	2.00 (4.18)
T3_2	50.39	48.85	53.55	92.11	45.99 (94.15)	43.97 (90.00)	2.03 (4.15)
T3_3	52.27	50.63	53.54	91.99	48.55 (95.90)	46.31 (91.48)	2.24 (4.42)
T4_1	47.93	46.45	53.98	92.03	42.07 (90.57)	40.16 (86.46)	1.91 (4.12)
T4_2	54.22	52.56	53.71	91.94	49.33 (93.84)	46.82 (89.07)	2.51 (4.77)
T4_3	49.93	48.99	53.76	95.02	45.36 (92.58 )	43.23 (88.25)	2.12 (4.33)
T5_1	54.90	53.82	53.65	95.11	49.27 (91.55)	47.31 (87.90)	1.96 (3.65)
T5_2	50.12	49.15	53.74	94.93	44.30 (90.13)	41.96 (85.36)	2.34 (4.77)
T5_3	57.01	55.88	53.86	95.15	51.39 (91.97)	48.92 (87.53)	2.48 (4.43)

Notes: T1, T2, T3, T4, and T5 stand for five developmental stages (7, 14, 21, 28, and 35 DAA) during grain filling. _1, _2, and _3 represent the three biological replicates. M represents million. GC Content represents the percentage of guanine and cytosine in the clean reads. Q30 represents the percentage of nucleotides with a quality value ≥ 30.

## Supplementary Materials

Supplementary materials can be found at https://www.mdpi.com/1422-0067/21/14/5031/s1.

## Abbreviations

ABA	Abscisic acid
bHLH	Basic helix-loop-helix
BR	Brassinolide
bZIP	Basic leucine zipper
CTK	Cytokinin
DAA	Days after anthesis
DEGs	Differentially expressed genes
ERF	Ethylene responsive factor
ETH	Ethylene
FPKM	Fragments Per kb Per Million Reads
GO	Gene Ontology
IAA	Auxin
JA	Jasmonic acid
KEGG	Kyoto Encyclopedia of Genes and Genomes
MYB	V-myb avian myeloblastosis viral oncogene homolog
NAC	NAM-ATAF1-2-CUC2
NF-YB1	Nuclear Factor Y B1
qRT-PCR	Quantitative real-time polymerase chain reaction
SA	Salicylic acid
TFs	Transcription factors
TKW	Thousand kernel weight
WRKY	WRKY domain transcription factors
